# Prior Practice Affects Movement-Related Beta Modulation and Quiet Wake Restores It to Baseline

**DOI:** 10.3389/fnsys.2020.00061

**Published:** 2020-08-18

**Authors:** Elisa Tatti, Serena Ricci, Aaron B. Nelson, Dave Mathew, Henry Chen, Angelo Quartarone, Chiara Cirelli, Giulio Tononi, Maria Felice Ghilardi

**Affiliations:** ^1^CUNY School of Medicine, The City University of New York, New York, NY, United States; ^2^Department of Biomedical, Dental, Morphological and Functional Imaging Sciences, University of Messina, Messina, Italy; ^3^Department of Psychiatry, University of Wisconsin-Madison, Madison, WI, United States

**Keywords:** EEG, beta, oscillations, ERD/ERS, motor control

## Abstract

Beta oscillations (13.5−25 Hz) over the sensorimotor areas are characterized by a power decrease during movement execution (event-related desynchronization, ERD) and a sharp rebound after the movement end (event-related synchronization, ERS). In previous studies, we demonstrated that movement-related beta modulation depth (peak ERS-ERD) during reaching increases within 1-h practice. This increase may represent plasticity processes within the sensorimotor network. If so, beta modulation during a reaching test should be affected by previous learning activity that engages the sensorimotor system but not by learning involving other systems. We thus recorded high-density EEG activity in a group of healthy subjects performing three 45-min blocks of motor adaptation task to a visually rotated display (ROT) and in another performing three blocks of visual sequence-learning (VSEQ). Each block of either ROT or VSEQ was followed by a simple reaching test (*mov*) without rotation. We found that beta modulation depth increased with practice across *mov* tests. However, such an increase was greater in the group performing ROT over both the left and frontal areas previously involved in ROT. Importantly, beta modulation values returned to baseline values after a 90-min of either nap or quiet wake. These results show that previous practice leaves a trace in movement-related beta modulation and therefore such increases are cumulative. Furthermore, as sleep is not necessary to bring beta modulation values to baseline, they could reflect local increases of neuronal activity and decrease of energy and supplies.

## Introduction

Oscillations in the beta frequency range (13.5−25 Hz) are prominent in the entire sensorimotor network and show solid dynamics: desynchronization of beta power occurs with movement initiation (event-related desynchronization, ERD), while beta power rebounds (event-related synchronization, ERS) after the movement end (Lopes da Silva and Pfurtscheller, 1999; Neuper et al., 2006). Beta oscillations should function as a gatekeeper to sensorimotor information, with beta ERD representing a release of the sensorimotor network from the inhibitory power of beta activity (Lopes da Silva and Pfurtscheller, 1999; Toma, 2002; Kilavik et al., 2013). The amplitude of beta rebound is modulated by factors such as motor learning (Boonstra et al., 2007; Tan et al., 2016) and practice (Moisello et al., 2015; Nelson et al., 2017). In particular, recent studies from our laboratory have shown that extended practice in a reaching task is associated with a significant increase of the beta ERD-ERS peak-to-peak amplitude (i.e., beta modulation depth) over parietal and frontal regions. This measure is independent from mean power changes and is mostly due to amplitude increases of the peak ERS (Moisello et al., 2015; Nelson et al., 2017; Ricci et al., 2019a; Tatti et al., 2019). Interestingly, the beta modulation increase during the task is followed by a local beta power increase during the resting EEG (Moisello et al., 2015) and returns to baseline values when tested 24 h later (Nelson et al., 2017). In line with these observations, other works have demonstrated that beta ERS amplitude can be reduced by a sensorimotor perturbation (Boonstra et al., 2007) and enhanced after successful motor adaptation (Tan et al., 2016). The practice-related increase of beta modulation may thus represent use-dependent phenomena. If this were the case, first, previous learning activity that engages the sensorimotor system should be reflected in a progressive increase of beta modulation during successive performance in a simple motor test. Importantly, such an increase should be less evident after a learning task that does not involve the sensorimotor system. Second, if beta modulation enhancement was primarily caused by increased neuronal activity or temporary decrease of energy supply, a period of rest in quiet wake should restore beta modulation to baseline values. Conversely, if it were expression of long-term plasticity-related processes, a period of sleep would be needed for its renormalization. Therefore, we analyzed the changes of movement-related beta modulation depth during reaching tests (*mov*) recorded after three 1-h blocks of either a visuo-motor adaptation task (ROT) or a visual sequence learning task (VSEQ). After the third block of practice, one group of subjects took a 90-min nap and another rested quietly without sleeping for the same period of time. We found that, while beta modulation depth increased progressively across *mov* tests, the group performing ROT displayed greater values than the VSEQ group.

## Materials and Methods

### Subjects and Experimental Design

Two groups of right-handed healthy subjects with normal or corrected vision were enrolled for this study. During the morning hours, 28 participants (mean age ± SD: 24.4 ± 4.0 years, 16 women) performed three blocks with a visuo-motor adaptation task (ROT) and 23 subjects (mean age ± SD: 23.3 ± 4.6 years, 12 women) three blocks of visual sequence learning task (VSEQ).

**FIGURE 1:**
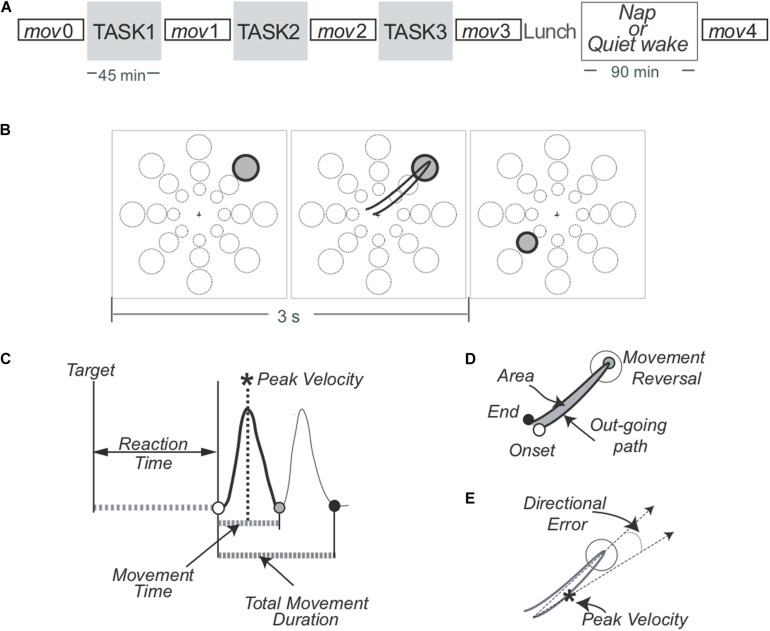
**(A)** Experimental design. Four blocks of a simple reaching task (*mov*) were interspaced by three 45-min blocks of practice of either ROT, an implicit motor learning task, or VSEQ, a visual working memory task with a declarative learning component. Another *mov* (*mov*4) was performed after lunch and a 90-min interval of either nap or quiet rest. **(B)** *Mov* test. One of 24 targets (three distances, eight directions) appeared in unpredictable order every 3 s. **(C–E)** Performance measures related to the movement.

All participants had no history of sleep or medical disorders. They reported an average of 7−8 h/night sleep for at least a week before the experiment, with consistent bed and rise times, as verified by their sleep diaries. Alcohol and caffeine-containing beverages were not allowed starting the night before and throughout each experiment. Briefly, around 8 am, subjects were fitted with a 256-channel HydroCel Geodesic Sensor Net (Electrical Geodesics Inc., Eugene, OR, United States). Participants were seated in a sound-shielded room in front of a computer display. As outlined in Figure 1A, in both ROT and VSEQ sessions, a baseline assessment (*mov*0) was run before both experimental conditions. Then, they performed three 45-min blocks of ROT, an implicit motor learning task (see description below), or VSEQ, a visual working memory task with a declarative learning component (see description below). After each 45-min block, participants completed a *mov* test for a total of four *mov* tests. Each block lasted about 1 h. Finally, after a brief lunch, all subjects were further divided in two groups: one group (15 subjects after ROT and 12 after VSEQ) was asked to take a nap and another (13 subjects after ROT and 11 after VSEQ) to rest quietly with eyes closed listening to an audio book. After such period of time, subjects were tested again with *mov* (*mov4)* (Figure 1A). The investigation was carried out in accordance with the latest version of the Declaration of Helsinki. The local Institutional Review Board approved the study and participants signed an IRB-approved consent form.

#### *mov* Test

The characteristics of this test have been detailed in a recent paper (Tatti et al., 2019) and are illustrated in Figure 1B.

Briefly, subjects performed reaching movements with their right hand by moving a cursor on a digitizing tablet to targets appearing on a screen. The central starting point and the cursor were always visible. One out of 24 targets (three distances, eight directions) appeared on the screen every 3 s in randomized order. Instructions were to reach the target with out-and-back movements as fast as possible, with overlapping strokes, without stops in the target circle and without corrections. In addition, subjects were asked to minimize movement time and avoid anticipation. Each *mov* test entailed 96 movements.

As detailed in previous publications (Ghilardi et al., 2000, 2003; Perfetti et al., 2011a), we computed several measures for each movement. In this study, we focused on: reaction time, (i.e., the time from target appearance to movement onset); movement time, (i.e., the duration of the outgoing movement); total movement time, (i.e., the duration of the out and back movement); amplitude of peak velocity of the out-going segment (Figure 1C); normalized hand path area, a measure of interjoint coordination (i.e., the ratio between the area delimited by the out and back path and the squared path length, Figure 1D); directional error at peak velocity, (i.e., the difference between the direction of target and that of the trajectory at the time of peak velocity, Figure 1E). For each set, we also computed the percentage of correct movements, i.e., movements with values of reaction time, normalized hand path area and directional error within two standard deviations of the *mov*0 mean. Movements with any of such measures outside two standard deviations and those rejected from EEG preprocessing were excluded from EEG analyses.

#### ROT Task

Similarly to tasks used in previous studies (Huber et al., 2004; Perfetti et al., 2011a), in ROT we used an array of eight targets at a fixed distance in eight directions. One of targets blackened every 1.5 s in a random, unpredictable order. Instructions were the same as in *mov*. Participants performed three movement blocks of 21 sets of 56 movements each (30 s inter-set interval, 1176 movements per block). Differently from *mov*, every two sets, unbeknownst to the subjects, the direction of the cursor on the screen was rotated relative to the direction of the hand on the tablet to a maximum of 60°, in steps of 10°, 20°, or 30°. The small incremental rotation steps were implemented to minimize awareness and the use of cognitive strategies, thus triggering implicit learning processes. Importantly, the rotation steps were smaller in the first block and greater in the last one in order to keep a similar degree of adaptation across the entire session. Indeed, mean adaptation was similar in ROT1 (mean ± SD: 71.7% ± 4.2%), ROT2 (72.7% ± 3.7%), and ROT3 (71.9% ± 3.8%), suggesting that a similar learning rate occurred across ROT blocks. Importantly, each ROT block started and ended with two and three sets, respectively, without any imposed rotation; this was both to avoid and monitor possible interference of residual directional error on the subsequent *mov* test. Accordingly, we found that the mean directional error of the first and last set were similar in each block (mean ± SE: ROT1: 5.19 ± 0.16 vs. 5.49 ± 0.26; ROT2: 5.65 ± 0.23 vs. 5.36 ± 0.22; ROT3: 5.40 ± 0.17 vs. 5.64 ± 0.16; *F*_(1,27)_ = 0.36, *p* = 0.55), without significant differences across blocks (*F*_(2,54)_ = 0.72, *p* = 0.49) and Set × Block interaction (*F*_(2,54)_ = 1.68, *p* = 0.20). This indicates that, at the end of each ROT block, performance returned on average to baseline levels and thus, interference of residual directional errors (due to ROT after-effects) on the subsequent *mov* tests should be considered as minimal.

#### VSEQ Task

In this visual sequence learning task participants were asked to memorize several 12-element spatial sequences (Ghilardi et al., 2003, 2009; Moisello et al., 2013; Steinemann et al., 2016). Sequences appeared on the screen with targets blackening every 1.5 s. Each sequence was presented three times per set (36 target presentation/set). At the end of each set, subjects reported the sequence order verbally. The same sequence was repeated until the subject correctly reported it, and then a new sequence was presented. The average number of sets required to learn a sequence was used to assess the subjects’ learning rate in each 45-min block.

### EEG Recording and Analysis

High-density (HD) EEG data were acquired using a 257-channel HydroCel Geodesic Sensor Net (Electrical Geodesic Inc.) with a Net Amp 300 amplifier (250 Hz sampling rate, online reference electrode: Cz) and Net Station software (version 5.0). Sampling frequency was 250 Hz and channel impedances were maintained below 50 kΩ. All recorded data were preprocessed using EEGLAB v13.6.5b toolbox for MATLAB (v.2016b) (Delorme and Makeig, 2004; Makeig et al., 2004). The continuous signal was first filtered using a Finite Impulse Response Filter (FIR) between 1 and 80 Hz and Notch filtered at 60 Hz.

Recordings were then segmented in 4-s epochs centered on target onset and examined to remove sporadic artifacts and channels with poor signal quality. Additionally, Independent Component Analysis (ICA) with Principal Component Analysis (PCA)-based dimension reduction (max 108 components) was applied to identify stereotypical artifacts, such as eye movements and heartbeat. Electrodes with bad signal quality were reconstructed using spherical spline interpolation, whereas those located on the cheeks and neck were removed. Re-reference to overall signal average was finally applied on the resulting 180 channels.

### *mov* Test EEG

After preprocessing, we discarded *mov* test epochs corresponding to “wrong” movements (see *mov* test description above). After trial rejection, the average number of trials per subject for the ROT and VSEQ sessions was 69.81 (±18.25 SD) and 78.75 (±5.65 SD), respectively. Data were then time-locked to movement onset (−1 to 2.5 s). Fieldtrip-based time-frequency representations within the beta frequency range (13.5−25 Hz) were computed using Complex Morlet Wavelets (0.5 Hz bins, 10 cycles). Data were normalized by the average beta power of the entire epoch. Afterward, the beta ERS-ERD peak-to-peak difference (beta modulation depth) topography was computed for each subject on *mov*0 to identify the electrode with the maximum beta modulation depth and the six neighbor ones. Specifically, the peak ERD and ERS amplitude was first determined over three broad regions corresponding to the frontal, left, and right located channels; peak ERD was defined as the minimum value of beta power within an interval between 100 ms before movement onset to 950 ms after; ERS amplitude was the maximum value in the interval from 700 to 2500 ms. Those values were finally used to find the electrode with the maximum beta modulation depth (peak ERS-peak ERD) and the six neighbors (see Supplementary Figures S1, S2 for a topological representation of the channels selection for each participants). Importantly, from now on, we are going to refer to this electrodes selection as Frontal, Left, and Right Regions of Interest (ROIs).

Time-frequency analyses were carried out on the selected ROIs (1:55 Hz, 0.5 Hz bins, 3:10 wavelet cycles) and normalized by the total power of the baseline test (*mov*0) according to this formula: (*mov*_*n*_ – *mov*_0_)/*mov*_0_. Peak beta ERS, ERD, modulation depth magnitude, as well as the ERS and ERD peak timing values were finally computed.

#### Tasks EEG

Time-frequency analyses on the task EEG signal were run using the MATLAB Toolbox Fieldtrip (Oostenveld et al., 2011). Time varying spectral components were estimated convolving the signal with complex Morlet Wavelets at linearly spaced frequencies (1−55 Hz, 0.5 Hz bins) and increasing number of wavelets cycles (3:10 cycles). To determine practice-related changes during ROT1, epochs recorded during the first (F) and last (L) sets of movements (both without imposed rotation) were normalized by the total power of the first block (*all*) according to the following formula: (Task_*F/L*_−Task1*_*all*_*)/Task1*_*all*_*. The same approach was used for VSEQ, where the recordings of the first and the last sequence were used.

#### Analysis of the Nap and Quiet Rest Periods

Using standard guidelines (Berry et al., 2017), EEG recorded during the nap and the quiet rest periods was scored for sleep stages by trained experimenters with an open source, MATLAB-based, toolbox (Mensen et al., 2016). An experienced sleep scorer (AN) confirmed the scoring. Recordings were scored in 30-s epochs as: wakefulness (W), NREM sleep stage 1 (N1), NREM sleep stage 2 (N2), and NREM sleep stage 3 (N3). REM sleep was not present in either group. A mastoid reference was used and states were determined from classical derivations from the 10 to 20 montage (F4, F3, C4, C3, P3, P4, O1, and O2). The disappearance of posterior alpha oscillations and other rhythms associated with wakefulness as well as the occurrence of slow rolling eye movements were indicative of the transition to N1. Transition to N2 was marked by K complexes and sleep spindles, while transition and maintenance of N3 was determined by the occurrence of >75 uV slow waves for more than 20% of the epoch.

### Statistical Analyses

Non-parametric permutation statistics were run to identify significant practice-related changes (Last-First) in beta oscillatory activity during the first block of ROT and VSEQ task. The reference distribution was created using the Monte Carlo method with 10000 permutations. The false-alarm rate was controlled by applying cluster-correction under each permutation distribution, with a threshold of three significant channels to form a cluster and a critical alpha of 0.05 (Maris and Oostenveld, 2007).

Analyses on *mov* peak ERD, ERS, modulation depth, latency timings and average beta amplitude were conducted on IBM SPSS statistics v.25.

We ascertained the effect of extensive practice on *mov* beta modulation depth, peak ERS and ERD magnitude, timings, and average beta amplitude using a mixed-model repeated measure ANOVAs with the three ROIs (left, frontal and right) and the four morning blocks (*mov*0, *mov*1, *mov*2, and *mov*3) as within-subjects factors and with Task (ROT and VSEQ) as between-subjects factor. The same approach was used to test the effects of nap and quiet wake with three blocks (*mov*0, *mov*3, and *mov*4) and the left and frontal regions as within-subject factors, and with task (VSEQ and ROT) and Nap and Quiet wake groups as between-subjects factors. For all these analyses, violation of sphericity was addressed with Greenhouse-Geisser correction. All *post hoc* pairwise comparisons that followed significant main effects were Bonferroni corrected.

## Results

### Learning in ROT and VSEQ Show Different EEG Correlates

All the subjects completed the three blocks of either VSEQ or ROT and the *mov* tests without difficulty. The performance of the subjects during both sessions showed signs of learning. In ROT, subjects successfully adapted their movements to the imposed rotation, learning more than 70% of the imposed rotation in each ROT block (see methods). In addition, the interjoint coordination improved from ROT1 to ROT3, as shown by a decrement of the hand path area (ROT1, mean ± SD: 0.057 ± 0.013; ROT3, 0.049 ± 0.009; *t*_(27)_ = 3.60; *p* = 0.0006), suggesting the occurrence of meta-learning or a “learning how to learn” effect. Comparison of the recordings of the last and first set of movements in ROT1, both without imposed rotation, showed a power increase in the beta range in channels located over a broad region including the left temporo-parietal and frontal areas (22.4% ± 27.8%, cluster t: 138.45, *p* = 0.0003).

**FIGURE 2:**
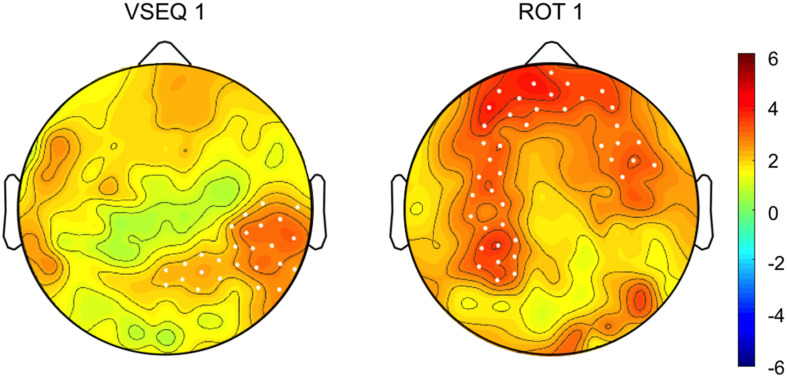
(Top) Topographic distribution of the difference in beta oscillatory activity between the last and first set/sequence VSEQ 1 (left) and ROT 1(right). (Bottom) Cluster *t*-values map. Dots indicate significant clusters of electrodes (*p* ≤ 0.05).

In VSEQ, the rate of learning improved from an average of 3.094 sets/sequence (SD: 0.667) in VSEQ1 to an average of 2.864 (SD: 0.716) in VSEQ3 (two-tailed paired *t*-test: *t*_(22)_ = 1.90; *p* = 0.036), suggesting that subjects improved their ability to learn sequences. Cluster-based permutation analysis in VSEQ1 (last vs. first sequence) revealed a significant increase of beta power in a cluster of electrodes over the right temporo-parietal area (11.2% ± 17.9% cluster t: 65.01, *p* = 0.033) (Figure 2).

In summary, in both ROT and VSEQ beta power increased during learning; however, beta power increases in VSEQ and ROT was observed in different channels, with increases over regions associated to motor practice during ROT.

### Previous Motor but Not Visual Learning Impairs Motor Performance in *mov*

**FIGURE 3:**
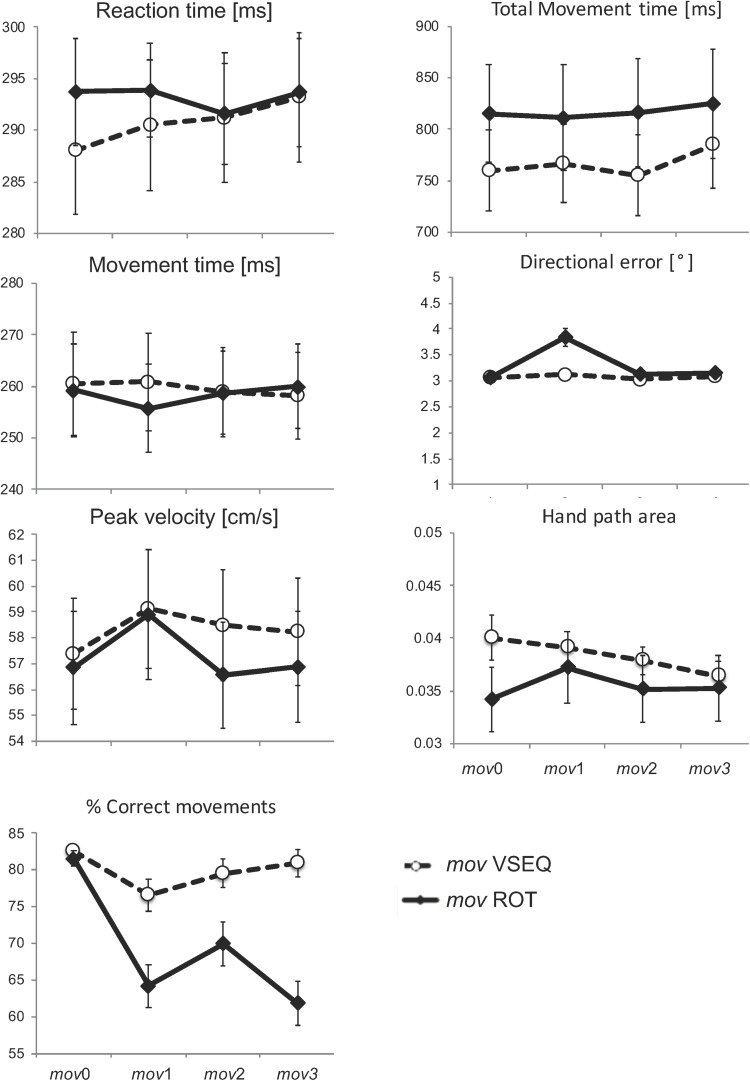
Mean and SE of performance measures for the correct movements in *mov*. No significant changes were observed across blocks and between practice groups for the kinematic measures.

Movements in *mov* were mostly straight and had bell-shaped velocity profiles both after VSEQ and ROT blocks. The percentage of correct movements decreased across blocks (*F*_(3,147)_ = 15.82, *p* < 0.00001) and differently affected the two tasks (F_(1,49)_ = 6.92, *p* = 0.011; block × task interaction: *F*_(3,1)_ = 6.19, *p* = 0.001). In fact, correct movements significantly decreased from *mov*0 to *mov*1 (*t*_(27)_ = 6.07; *p* < 0.0001), *mov*2 (*t*_(27)_ = 4.12; *p* = 0.0018), and *mov*3 (*t*_(27)_ = 6.88; *p* < 0.0001) only in ROT. In VSEQ, we found a significant difference only between *mov*0 and *mov*1 (*t*_(22)_ = 3.044; *p* = 0.036). Reaction time, movement time, peak velocity, total movement duration, hand path area, and directional error of the corrected movements were similar in the two groups and without significant changes across blocks (Figure 3 and Table 1).

**TABLE 1:** Results of mixed model ANOVAs for kinematic measures.

	Blocks			Blocks × Task		Task	
	*df*	*F*	*p*	*F* (49)	*p*	*F* (1)	*p*
Total Mov. Time	2.30	0.98	0.40	0.31	0.82	0.59	0.44
Movement Time	2.13	0.12	0.90	0.59	0.62	0.01	0.92
Peak Velocity	2.56	1.74	0.16	0.37	0.78	0.12	0.74
Reaction Time	2.54	1.02	0.39	1.38	0.25	0.11	0.75
Hand Path Area	2.54	2.01	0.13	2.69	0.06	0.28	0.60
Mean Dir. Error	2.87	1.43	0.24	0.27	0.84	1.14	0.29

In summary, the small but significant decrease of correct movements after the ROT blocks but not after the VSEQ blocks supports the notion that error rate increased only when the practiced task shared some characteristics with *mov*. Nevertheless, the kinematic features of the *mov* correct movements were similar in both groups and were not significantly influenced by the preceding task.

### Changes During *mov* Are Specific to the Previous Practice and the ROIs

As described in the methods, the selection of the ROIs to compute beta ERD, ERS and modulation depth were based on the topographical maps of *mov*0, recorded at baseline before either VSEQ or ROT learning task.

**FIGURE 4:**
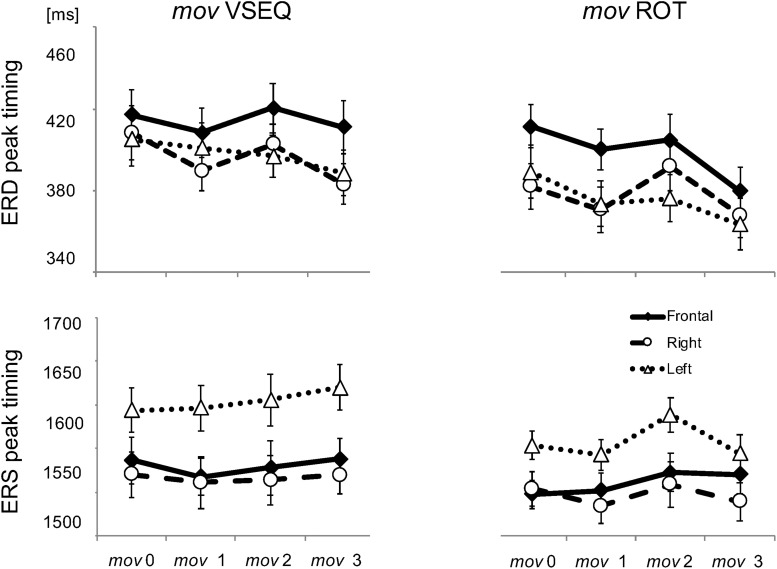
Mean and SE of beta ERD and ERS peak timings in the Frontal, Left and Right ROIs.

We first focus on the changes of ERD and ERS peak timings, and then on those of amplitudes of beta modulation, ERS and ERD. Importantly, analyses were performed on the data corresponding to the correct movements only in order to avoid possible contamination resulting from the inclusion of wrong movements. The results of the main mixed-model ANOVAs and *post hoc* comparisons are reported in Tables 2, 3. Briefly, the timings of ERS and ERD peaks differed in the three ROIs, independently of the practiced task. On average, peak ERD over the frontal ROI occurred 20 ms later than over the other regions, whereas peak ERS over the left ROI occurred 53 ms later than over the other two ROIs. The ERD timing decreased across blocks, peaking earlier in *mov3* compared to *mov2* (16 ± 0.5 ms, *p* = 0.006) and *mov*0 (20 ± 0.7 ms, *p* = 0.022) (Figure 4 and Table 3).

**TABLE 2:** Results of mixed model ANOVAs for the beta modulation depth, peak beta ERS, ERD, and average beta amplitude, and the timing of the peak ERD and ERS.

		Modulation depth	ERS amplitude	ERD amplitude	ERS timing	ERD timing	Mean power
Blocks	*df*	1.4, 68.96	1.39, 68.29	1.38, 67.84	2.81, 137.7	2.45, 120.0	1.26, 61.78
	*F*	22.99	22.79	16.68	1.21	4.69	16.39
	*p*	**<0.0001**	**<0.0001**	**<0.0001**	0.31	**0.007**	**<0.0001**
	η^2^p	0.32	0.32	0.25	0.02	0.09	0.25
ROI	*df*	1.61, 2.54	1.60, 78.32	1.9, 96.61	1.88, 91.92	1.93, 94.46	1.62, 79.32
	*F*	9.05	7.75	2.86	25.47	11.10	2.02
	*p*	0.001	**0.002**	0.063	**<0.0001**	**<0.0001**	0.149
	η^2^p	0.16	0.14	0.06	0.34	0.19	0.04
Task	*df*	1, 49	1, 49	1, 49	1, 49	1, 49	1, 49
	*F*	5.47	5.45	0.73	1.32	2.52	1.19
	*p*	**0.023**	**0.024**	0.400	0.256	0.119	0.282
	η^2^p	0.10	0.10	0.02	0.26	0.05	0.02
Blocks × Task	*df*	1.41, 68.96	1.39, 68.29	1.38, 68.83	2.81, 137.7	2.45, 120.0	1.26, 61.78
	*F*	2.71	2.63	0.54	0.91	0.21	1.06
	*p*	**0.047**	0.097	0.524	0.431	0.853	0.325
	η^2^p	0.05	0.05	0.01	0.02	0.00	0.02
ROI × Task	*df*	1.63,78.98	1.60, 78.33	1.97, 96.61	1.88, 91.92	1.93, 94.46	1.62, 79.32
	*F*	1.63	1.45	1.46	1.00	0.28	2.20
	*p*	0.206	0.241	0.237	0.366	0.750	0.128
	η^2^p	0.03	0.03	0.03	0.02	0.01	0.04
ROI × Block	*df*	1.94, 95.03	1.96, 95.99	2.81, 137.6	5, 244.97	4.88, 239.2	2.08, 102.1
	*F*	3.62	4.03	0.41	0.58	0.53	1.51
	*p*	**0.032**	0.052	0.730	0.719	0.784	0.226
	η^2^p	0.07	0.06	0.01	0.01	0.01	0.03
ROI × Block × Task	*df*	1.94, 95.03	1.96, 95.99	2.81, 137.6	5, 244.97	4.88, 239.2	2.08, 102.1
	*F*	1.25	1.31	0.30	0.69	0.73	1.73
	*p*	0.292	0.270	0.813	0.630	0.600	0.181
	η^2^p	0.03	0.03	0.01	0.01	0.02	0.03

*Bold values denote statistically significant results at the p < 0.05 level.*

**TABLE 3:** Bonferroni-corrected *post hoc* comparisons for the magnitude of beta ERD and ERS and modulation depth, peak ERS and ERD timing, and average beta power.

		ROIs			Blocks					
		Left Right	Right Front	Front Left	Mov 0 Mov1	Mov 0 Mov 2	Mov 0 Mov 3	Mov 1 Mov 2	Mov 1 Mov 3	Mov 2 Mov 3
Beta modulation depth	Mean diff.	0.59	−0.41	−0.18	−0.39	−0.71	−0.85	−0.32	−0.46	−0.14
	*SE*	0.15	0.11	0.17	0.08	0.12	0.17	0.07	0.12	0.08
	*p*	**0.001**	**0.001**	0.824	**<0.001**	**<0.001**	**<0.001**	**<0.001**	**0.001**	0.622
ERS	Mean diff.	0.57	−0.39	−0.18	−0.41	−0.74	−0.90	−0.33	−0.49	−0.16
	*SE*	0.16	0.11	0.18	0.09	0.12	0.18	0.07	0.12	0.09
	*p*	**0.002**	**0.002**	0.936	**<0.001**	**<0.001**	**<0.001**	**<0.001**	**0.001**	0.417
ERD	Mean diff.	−0.03	0.01	0.01	−0.02	−0.04	−0.06	−0.02	−0.04	−0.02
	*SE*	0.01	0.01	0.01	0.01	0.01	0.01	0.01	0.01	0.01
	*p*	0.066	0.630	0.776	**0.026**	**0.002**	**<0.001**	**0.019**	**0.001**	**0.001**
Timing ERS	Mean diff.	0.06	−0.01	−0.05	0.01	−0.01	0.00	−0.02	−0.01	0.01
	*SE*	0.01	0.01	0.01	0.01	0.01	0.01	0.01	0.01	0.01
	*p*	<0.001	0.632	**<0.001**	1.000	1.000	1.000	0.333	1.000	1.000
Timing ERD	Mean diff.	0.00	−0.02	0.02	0.01	0.00	0.02	−0.01	0.01	0.02
	*SE*	0.01	0.01	0.01	0.01	0.01	0.01	0.01	0.01	0.01
	*p*	1.000	**0.002**	**<0.001**	0.345	1.000	**0.022**	0.567	1.000	**0.006**
Average Beta	Mean diff.	0.09	0.02	−0.07	−0.10	−0.21	−0.26	−0.10	−0.12	−0.05
	*SE*	0.05	0.05	0.03	0.03	0.04	0.06	0.03	0.04	0.03
	*p*	0.263	1.000	0.154	**0.002**	**<0.001**	**0.001**	**0.001**	**0.003**	0.307

*Bold values denote statistically significant results at the p < 0.05 level.*

**FIGURE 5:**
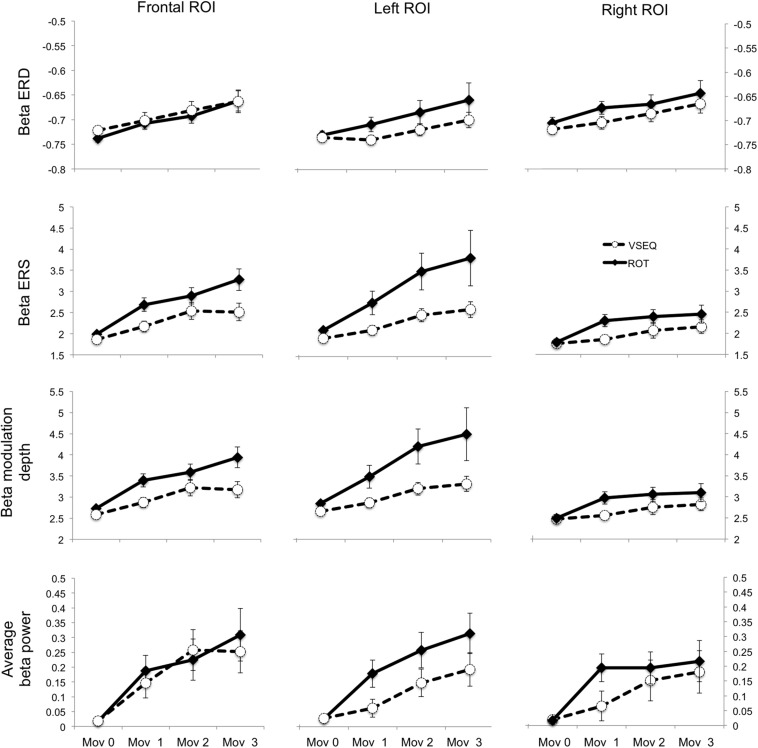
Mean and SE of the magnitude of beta ERD, ERS, modulation depth, and average beta (dimensionless) in the Frontal, Left and Right ROIs.

**FIGURE 6:**
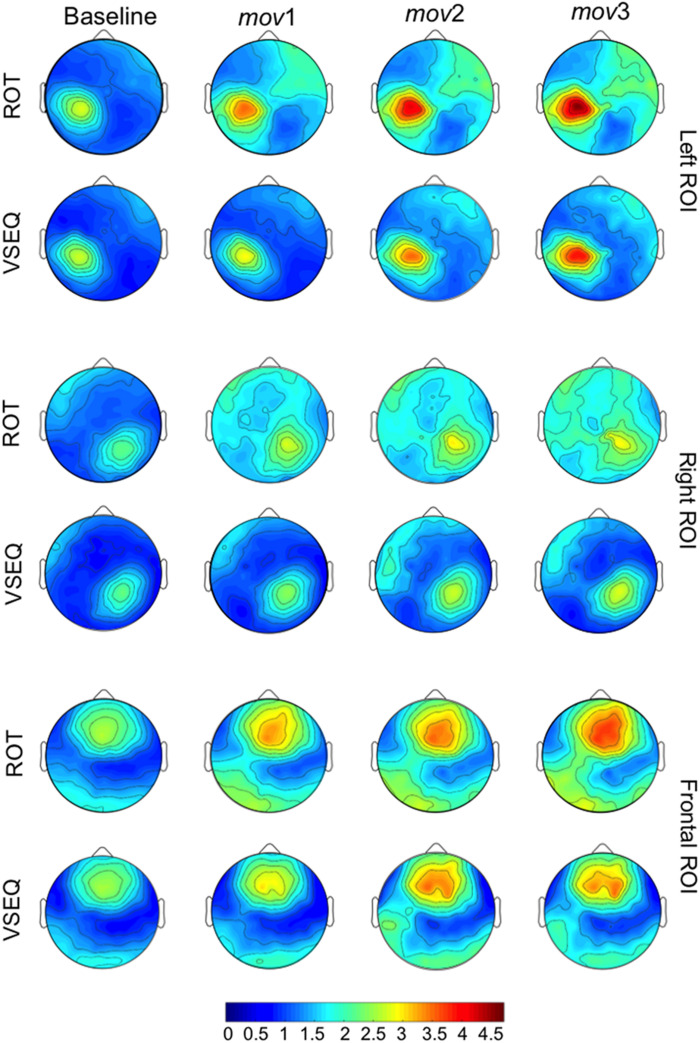
Average *mov* VSEQ (top) and *mov* ROT (bottom) beta modulation depth topographies for the Left, Right, and Frontal ROIs.

ANOVAs on beta modulation depth revealed effects of ROI, block, and task. Similar results were found for ERS, but not for ERD magnitude (Tables 2, 3). As in a previous work (Moisello et al., 2015), beta modulation depth increased across blocks (Tables 2, 3) and was greater over the left ROI, followed by the frontal and the right ROIs (Figures 5, 6). In both ROT and VSEQ sessions, the right ROI reached significantly lower values than the other two ROIs (Table 3). The magnitude of beta modulation was different in the two sessions: greater values were found when *mov* was preceded by ROT than by VSEQ (Figures 5, 6 and Table 2).

Separate analyses for each ROI (Table 4) revealed a greater beta modulation depth after ROT than after VSEQ for the frontal and left ROIs, but not for the right ROI. Importantly, despite mean beta amplitude increased across the four blocks, we did not find differences between ROIs and tasks (Table 2).

**TABLE 4:** Results of mixed model ANOVAs for the magnitude of beta ERD and ERS, and modulation depth for each ROI.

		Modulation depth			ERS Amplitude			ERD Amplitude		
		Left	Frontal	Right	Left	Frontal	Right	Left	Frontal	Right
Block	*df*	1.15,56.5	1.98,96.9	1.98,97.2	1.15,56.5	14, 95.0	1.93,94.6	1.22,59.7	1.73,84.5	1.48,72.8
	*F*	12.192	24.022	10.581	11.47	24.33	11.39	6.36	16.46	9.04
	*p*	**0.001**	**<0.0001**	**<0.0001**	0.001	**<0.0001**	**<0.0001**	**0.01**	**<0.0001**	**0.001**
	η^2^p	0.200	0.330	0.178	0.190	0.330	0.190	0.115	0.250	0.156
Task	*df*	1, 49	1, 49	1, 49	1, 49	1, 49	1, 49	1, 49	1, 49	1, 49
	*F*	4.102	5.426	1.967	3.9	4.96	2.35	1.61	0.17	1.05
	*p*	**0.048**	**0.024**	0.167	0.054	**0.031**	0.13	0.21	0.68	0.311
	η^2^p	0.077	0.100	0.039	0.07	0.09	0.05	0.032	0.004	0.021
Block × Task	*df*	1.15,56.5	1.98,96.9	1.98,97.2	1.15,56.5	14, 95.0	1.93,94.6	1.22,59.7	1.73,84.5	1.48,72.8
	*F*	1.97	2.63	1.61	2.02	2.45	1.53	0.68	0.27	0.21
	*p*	0.164	0.08	0.205	0.16	0.093	0.22	0.44	0.73	0.747
	η^2^p	0.040	0.051	0.032	0.040	0.050	0.030	0.010	0.005	0.004

*Bold values denote statistically significant results at the p < 0.05 level.*

In summary, these results confirm that beta modulation depth increases with practice. The increase is more evident over the left and the frontal ROIs following a motor learning task, ROT, which previously engaged such areas.

### Both Nap and Quiet Rest Restore Beta Modulation Depth to Baseline Values

We then ascertained whether a period of either nap or quiet wake changed performance and beta modulation values. Therefore, after the three morning blocks of practice in VSEQ and ROT, a group of subjects took a nap while another group rested quietly but without sleeping for 90 min. The nap group after VSEQ (*N* = 12) slept for an average of 75% (SE: 5%) of the time not differently from the group that napped after ROT (*N* = 15, 76% ± 4%; two-tailed t-test for independent means: *t*_25_ = 0.13, *p* = 0.45, (μ1−μ2) = 1.2). NREM stage N2 was evident in all the subjects (average time in N2: after VSEQ: 35% ± 4%, after ROT: 37% ± 3%; *t*_25_ = 0.33, p = 0.37, (μ1−μ2) = 1.72), while N3 was present in 10 subjects after VSEQ (average time: 23% ± 7%) and in 12 after ROT (29% ± 5%; *t*_20_ = 0.75, *p* = 0.23, (μ1−μ2) = 6.07). This indicates that sleep was equally consolidated and deep after both VSEQ and ROT. Importantly, no differences between VSEQ and ROT conditions were observed in both delta (1−4 Hz) and theta (4.5−8 Hz) amplitude in the ROIs showing increased beta modulation (see Supplementary Figure S3). In the quiet wake group, five subjects out of 24 reached N1 stage (VSEQ, *N* = 3 out of 11, average time: 9% ± 4%; ROT, *N* = 2 out of 13, average time: 9% ± 2%) and two of them (one in VSEQ and the other in ROT) reached N2 stage but only for a short period of time (both subjects: 10%).

**FIGURE 7:**
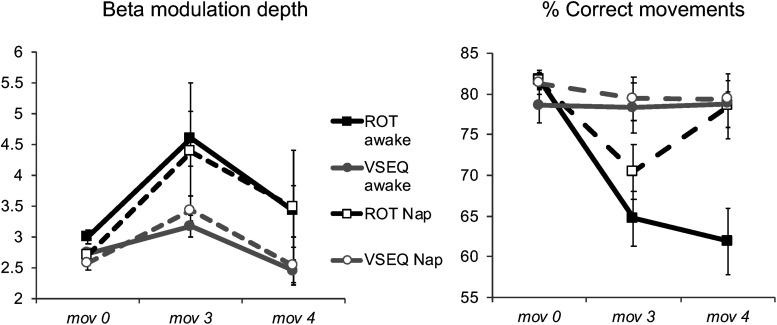
(Left) Mean and SE of beta modulation depth (dimensionless) over the Left ROI after 90 min of either Nap or Quiet wake (Blocks: *mov*0, *mov*3, and *mov*4), in both ROT and VSEQ groups. (Right) Number of correct *mov* movements (%) during the baseline (*mov*0), the last *mov* of the morning (*mov*3), and after 90 min of either Nap or Quiet Wake (*mov*4).

We first compared the number of correct movements in *mov*4, recorded after the 90-min interval, with *mov*0, recorded in the morning at baseline, and *mov*3, recorded at the end of the morning blocks, with a mixed model ANOVA (Block as repeated-measure factor, Task, and Group as between-subjects factors) followed by two one-way ANOVAs to isolate the effects for ROT and VSEQ (Table 5). Briefly, such analyses revealed that *mov* performance that was degraded in *mov*3 after ROT (Figure 3) improved in *mov*4 reaching baseline levels only after a nap but not after quiet wake (Figure 7, ROT awake vs. nap, *mov*0: mean difference = 2.01 ± 1.42, *p* = 0.165; *mov*3: mean difference = −6.18, *p* = 0.151; *mov*4: mean difference = −14.94 ± 4.18, *p* = 0.001). The increase of correct movements from *mov3* to *mov4* was positively correlated with delta power in the frontal ROI during the nap (*N* = 11, N2: *r* = 0.629, *p* = 0.038, 95% CI [0.047, 0.892]; *N* = 8, N3: *r* = 0.865, *p* = 0.005, 95% CI [0.411, 0.975]). Performance in *mov*4 after VSEQ (Figure 7) remained at the same high levels of the previous testing times (Figure 3) both after nap and quiet wake.

**TABLE 5:** (Left) Results of mixed model ANOVAs for the % of correct movements in mov0, mov3, and mov4 as Blocks, ROT vs. VSEQ as Task, and Nap vs. Awake as Group. (Right) Results of mixed model ANOVA for the ROT Task condition only.

	% Correct movements			
	*df*	*F*	*p*	η^2^p
Block	1.874 88.059	17.786	**<0.0001**	0.275
Task	1 47	10.004	**0.003**	0.175
Group	1 47	5.261	**0.026**	0.101
Group × Task	1 47	2.322	0.134	0.047
Block × Group	1.87 88.06	3.847	**0.027**	0.076
Block × Task	1.87 88.06	13.133	**<0.0001**	0.218
Block × Group × Task	1.87 88.06	6.187	0.003	0.116

	**ROT % Correct movements**			
	***df***	**F**	**p**	**η^2^p**

Block	1.608 54.665	25.769	**<0.0001**	0.431
Group	134	5.92	**0.02**	0.148
Block × Group	1.608 54.665	8.607	0.001	0.202

*Bold values denote statistically significant results at the p < 0.05 level.*

We finally compared the magnitude of beta modulation in *mov*4, *mov*0 and *mov*3. The results of the ANOVA (Table 6) revealed only a significant effect of block and a borderline effect of task. Importantly, *post hoc* tests showed that, following the morning increase (*mov*3 vs. mov0: mean difference = 1.01 ± 0.22, *p* < 0.0001), beta modulation depth decreased after the 90-min interval (*mov*4 vs. *mov*3: mean difference = 0.91 ± 0.09, *p* < 0.00001), reaching baseline values of *mov*0 (*mov*4 vs. *mov*0: mean difference = 0.10 ± 0.19, *p* = 1, Figure 7). We obtained similar results for the magnitude of ERD, ERS and mean beta power (Table 6). Interestingly, the decrease of beta modulation, ERS and ERD values in the two subjects of the awake group that reached N2 was smaller than the average of the awake group. Indeed, if sleep contributed to decrease beta modulation values, we would have expected greater decreases in these two subjects.

**TABLE 6:** Results of mixed model ANOVAs for the changes in the magnitude of beta ERD and ERS and modulation depth after 90 min of either Nap or Quiet wake (Blocks: *mov*0, *mov*3, *mov*4; Task: ROT, VSEQ; Group: Nap, Quiet wake).

		Modulation depth	ERS Amplitude	ERD Amplitude	Average Beta
Block	*df*	1.26, 59.4	1.26, 59.4	1.44, 67.7	1.26, 59.29
	*F*	19.998	19.536	9.811	10.69
	*p*	**<0.0001**	**<0.0001**	0.001	**0.001**
	η^2^p	0.298	0.294	0.173	0.185
ROI	*df*	1, 47	1, 47	1, 47	1, 47
	*F*	2.485	1.996	0.766	0.455
	*p*	0.122	0.164	0.386	0.503
	η^2^p	0.05	0.041	0.016	0.01
Task	*df*	1, 47	1, 47	1, 47	1, 47
	*F*	3.688	3.542	6.629	1.104
	*p*	0.061	0.066	**0.013**	0.299
	η^2^p	0.073	0.07	0.124	0.023
Group	*df*	1, 47	1, 47	1, 47	1, 47
	*F*	0.447	0.65	0.464	2.394
	*p*	0.507	0.424	0.499	0.129
	η^2^p	0.009	0.014	0.01	0.048
Block × Task	*df*	1.26, 59.4	1.26, 59.4	1.44, 67.7	1.26, 59.29
	*F*	2.679	2.5	3.204	1.074
	*p*	0.099	0.112	0.062	0.346
	η^2^p	0.054	0.051	0.064	0.022
Block × Group	*df*	1.26, 59.4	1.26, 59.4	1.44, 67.7	1.26, 59.29
	*F*	1.207	1.346	0.757	2.041
	*p*	0.288	0.259	0.433	0.155
	η^2^p	0.025	0.028	0.016	0.042
ROI × Task	*df*	1, 47	1, 47	1, 47	1, 47
	*F*	1.514	1.566	0.242	2.05
	*p*	0.225	0.217	0.625	0.159
	η^2^p	0.031	0.032	0.005	0.042
ROI × Block	*df*	1.43, 67.3	1.42, 66.9	1.51, 71	1.24, 58.26
	*F*	0.677	0.484	0.374	0.291
	*p*	0.599	0.555	0.63	0.748
	η^2^p	0.499	0.01	0.008	0.006
Task × Group	*df*	1, 47	1, 47	1, 47	1, 47
	*F*	0.215	0.184	0.048	0.004
	*p*	0.645	0.67	0.827	0.951
	η^2^p	0.005	0.004	0.001	<0.001
ROI × Group	*df*	1, 47	1, 47	1, 47	1, 47
	*F*	2.152	0.484	1.814	0.194
	*p*	0.149	0.555	0.184	0.661
	η^2^p	0.044	0.01	0.037	0.004
Block × Task × Group	*df*	1.26, 59.4	1.26, 59.4	1.44, 67.7	1.26, 59.29
	*F*	0.378	0.396	0.626	0.186
	*p*	0.59	0.58	0.488	0.831
	η^2^p	0.008	0.008	0.013	0.004
ROI ×Task × Group	*df*	1, 47	1, 47	1, 47	1, 47
	*F*	0.046	0.094	2.137	0.822
	*p*	0.832	0.76	0.15	0.369
	η^2^p	0.001	0.002	0.043	0.017
ROI × Block × Task	*df*	1.43, 67.3	1.42, 66.9	1.51, 71	1.24, 58.26
	*F*	1.132	0.97	1.38	0.186
	*p*	1.001	0.358	0.255	0.726
	η^2^p	0.349	0.02	0.029	0.004
ROI × Block × Group	*df*	1.43, 67.3	1.42, 66.9	1.51, 71	1.24, 58.26
	*F*	0.445	0.489	0.42	0.112
	*p*	0.577	0.552	0.602	0.894
	η^2^p	0.009	0.01	0.009	0.002
ROI × Block × Group × Task	*df*	1.43, 67.3	1.42, 66.9	1.51, 71	1.24, 58.26
	*F*	0.128	0.142	0.526	0.679
	*p*	0.81	0.795	0.543	0.443
	η^2^p	0.003	0.003	0.011	0.014

*Bold values denote statistically significant results at the p < 0.05 level.*

These results suggest that, while sleep is necessary to restore performance, a period of quiet rest without sleep is enough to reestablish beta modulation depth to baseline levels.

## Discussion

The present study shows that increases of movement-related beta modulation depth are affected by former practice: in fact, they were greater after a visuo-motor learning task (ROT) than after a visual sequence-learning task (VSEQ). This task difference was observed over the left and frontal regions, areas that showed sustained increase of beta power during the ROT task. Crucially, we also found that beta modulation depth returned to the morning baseline values after a period of either quiet wake or sleep. Altogether, these findings suggest that former practice influences the magnitude of beta modulation increase, as a sort of performance “signature” that, however, is short lasting and does not require sleep to fade away. This implies that the practice-dependent increases may reflect increased neuronal activity or temporary decrease of energy supply, rather than full-fledged plasticity phenomena per se.

### Regional Differences of Beta ERD/ERS Dynamics

The increases of beta modulation depth were different in the three ROIs. Beta oscillations in the motor, somatosensory, and frontal regions likely reflect different aspects of motor planning and execution that involve sensory, motor, and cognitive processes. Indeed, beta desynchronization during movement preparation and execution (ERD) and its rebound after movement completion (ERS) are predominant in the sensorimotor region contralateral to the moving effector, but are also observed in the ipsilateral and frontal regions (Rektor et al., 2006; Zaepffel et al., 2013; Meziane et al., 2015; Moisello et al., 2015; Ricci et al., 2019b) as also shown in the present work. However, our results further show that, while ERD magnitude was similar across ROIs, beta ERS magnitude was greater over the left and frontal areas and significantly lower over the right ROI, where practice-related increase was minimal. The discrepant results between ROIs suggest that beta rebound is a phenomenon that mostly involves the contralateral sensorimotor and frontal areas and less so the ipsilateral area. This finding is in agreement with other results showing that ipsilateral sensorimotor activity is linked mainly to movement selection and planning (Rao et al., 1993; Haaland et al., 2004) rather than to the processes occurring after movement cessation. Also, in line with past studies (Ohara et al., 2000; Szurhaj et al., 2003; Parkes et al., 2006; Wilson et al., 2010; Moisello et al., 2015; Heinrichs-Graham et al., 2017), the present results highlight the contribution of the frontal region not just to motor planning (Tombini et al., 2009; Perfetti et al., 2011b) but also to feedforward processes that occur after the movement; these are mostly reflected in the ERS magnitude, and are essential for updating internal models and learning. Indeed, beta ERS has been associated with many frontal functions, such as the maintenance of sensorimotor and cognitive sets (Gilbertson et al., 2005; Pogosyan et al., 2009; Engel and Fries, 2010; Tan et al., 2014a, b, 2016), the processing of sensory reafference (Cassim et al., 2001; Alegre et al., 2002), top-down executive control (Buschman and Miller, 2007; Spitzer and Haegens, 2017), as well as visuomotor attention (Classen et al., 1998; Kilavik et al., 2013).

### Increases of Beta Modulation Depth Are Affected by Previous Practice

As in our previous work (Nelson et al., 2017; Ricci et al., 2019a, b), we found that beta modulation depth increased with practice over the three ROIs. Additionally, such increases were present, although to a lesser extent, after a visual learning task, a finding likely due to a carry-over effect from a *mov* test to the following one. This is in agreement with previous work showing that increased beta modulation can occur within a set with less than the 96 movements of each *mov* test (Nelson et al., 2017). The present results further demonstrate that such increases may be carried over to another test almost 1 h later, i.e., the time between two successive *mov* tests. The increases were mostly driven by beta ERS as discussed above, thus confirming that practice mainly affects the magnitude of the post-movement beta rebound.

A novel result is that beta modulation depth differently evolved after the two tasks. Starting from comparable baseline values, the magnitude beta modulation over the frontal and left parietal regions increased more when the test was preceded by the visuo-motor learning task (ROT). Indeed, while beta increases during the VSEQ task were confined to the right region, beta power during the ROT task increased locally over frontal and left parietal channels (Figure 3). The patterns of beta increase during the tasks indicate the occurrence of a cumulative effect of motor practice during the ROT task to beta modulation depth on successive *mov* tests. Therefore, the increases of beta modulation depth during *mov* may be the reflection of local neuronal use-dependent phenomena related to the development of the early phases of long-term potentiation. Support to this notion comes from studies in humans showing that intermittent theta burst stimulation produced an increase of cortical excitability (measured using MEPs) together with an increase of the amplitude of post-movement beta synchronization (Hsu et al., 2011). Moreover, since beta ERD likely reflects activation of the motor cortex and depression of the sensory cortex and beta ERS the reactivation of the sensory network (Lee and Schmit, 2018), the increase of beta modulation may express functional neural changes due to induction of long term potentiation by a repetitive pattern of activation and inactivation of the sensory and motor areas. Thus, the increases of beta modulation depth across blocks could represent the local and progressive saturation of the capacity for plasticity within the cortical areas involved in movement planning and execution (Moisello et al., 2015; Nelson et al., 2017).

### A Period of Quiet Rest Returns Beta Modulation Depth to Baseline Values

Previous studies using a TMS-based repetitive pattern of sensorimotor activation have shown that the amplitude of beta modulation may express functional neural changes linked to the induction of long-term plasticity (LTP) processes (Hsu et al., 2011). These results suggest that our practice-dependent increases of beta modulation depth could be expression of plasticity-related phenomena. The early phases of LTP are short lasting (minutes to hours) and can be induced in brain slice preparations with a weak protocol (Huang, 1998). Conversely, the achievement of later phases needs a stronger induction protocol, the synthesis of new protein (Muller et al., 2002) and the decay of LTP early phases (Xiao et al., 1996; Villarreal et al., 2002) with the final result of increase of synaptic strengthening. All LTP phases require great energy availability and demand for delivery of cellular supplies. There is now strong evidence that only sleep can return the system to baseline levels in terms of net synaptic strength and cellular homeostasis while enhancing skill formation, thus overcoming the cellular consequences of full-fledged LTP (Tononi and Cirelli, 2014). However, sleep was not required to restore movement-related beta modulation depth to baseline values. It is therefore tempting to speculate that the increases of beta modulation depth may parallel some mechanisms involved in the early induction of plasticity-related phenomena, as previously indicated (Nelson et al., 2017). Alternatively but not exclusively, such increases might signal transient depletion of energy resources and cellular supplies needed to maintain LTP processes, a situation that can be restored to baseline levels by a period of rest and do not necessarily require sleep (Tononi and Cirelli, 2014).

Interestingly, as noted above, in the morning VSEQ practice we did not find reduction of beta modulation depth between 1-h apart *mov* tests. It is thus possible that one hour may not be sufficient to restore completely neural resources; alternatively, it is possible that the addition of activity, even when involving other brain areas, might have delayed the recovery process. In any event, while the decrease of beta modulation depth occurred even after a quiet wake period, sleep was necessary to improve the rate of correct movements, as shown by the comparison of the group that napped and the one that quietly rested after the ROT task. The discrepancy between performance and beta modulation suggests that the changes of beta modulation depth per se do not purely reflect learning-related changes. Therefore, as discussed above, the increase of movement-related beta modulation during the practice may not parallel the induction of full-scale long-term potentiation with all its benefits on performance but may signal greater neural activity and reduced energy and supplies.

## Conclusion

In conclusion, these results confirmed our previous findings by showing that practice of reaching movements induces beta modulation depth increases. More importantly, they show that the magnitude of the increase is amplified by previous practice that involves similar brain areas and that a period of quiet wake without sleep can restore beta modulation to baseline values.

## Data Availability Statement

The raw data supporting the conclusions of this article will be made available by the authors, without undue reservation.

## Ethics Statement

The studies involving human participants were reviewed and approved by the CUNY, Institutional Review Board (IRB). The patients/participants provided their written informed consent to participate in this study.

## Author Contributions

MG, CC, and GT conceived and designed the study. ET, SR, and AN contributed to data acquisition. ET, MG, DM, HC, SR, and AN carried out the data analyses. ET, MG, AQ, CC, and GT interpreted the results and revised the manuscript. ET, MG, and AQ drafted the manuscript. All the authors agreed to be accountable for all aspects of the work in ensuring that questions related to the accuracy or integrity of any part of the work are appropriately investigated and resolved.

## Conflict of Interest

The authors declare that the research was conducted in the absence of any commercial or financial relationships that could be construed as a potential conflict of interest.

## Supplementary Material

FIGURE S1 Topographies showing the selected channels for the Left, Frontal, and Right ROIs for each subject in the ROT group.

FIGURE S2 Topographies showing the selected channels for the Left, Frontal, and Right ROIs for each subject in the VSEQ group.

FIGURE S3 Delta and theta topographies during N2 and N3 stages in the ROT and VSEQ groups.

TABLE S1 Results of mixed model ANOVAs for the delta and theta amplitudes in the selected ROIs during N2 and N3 stages in the ROT and VSEQ Nap groups.
