# Supplementary figures and images for: Prior Practice Affects Movement-Related Beta Modulation and Quiet Wake Restores It to Baseline
Source: Front Syst Neurosci. 2020 Aug 18;14:61. doi: 10.3389/fnsys.2020.00061 (PMC7462015; doi:10.3389/fnsys.2020.00061)

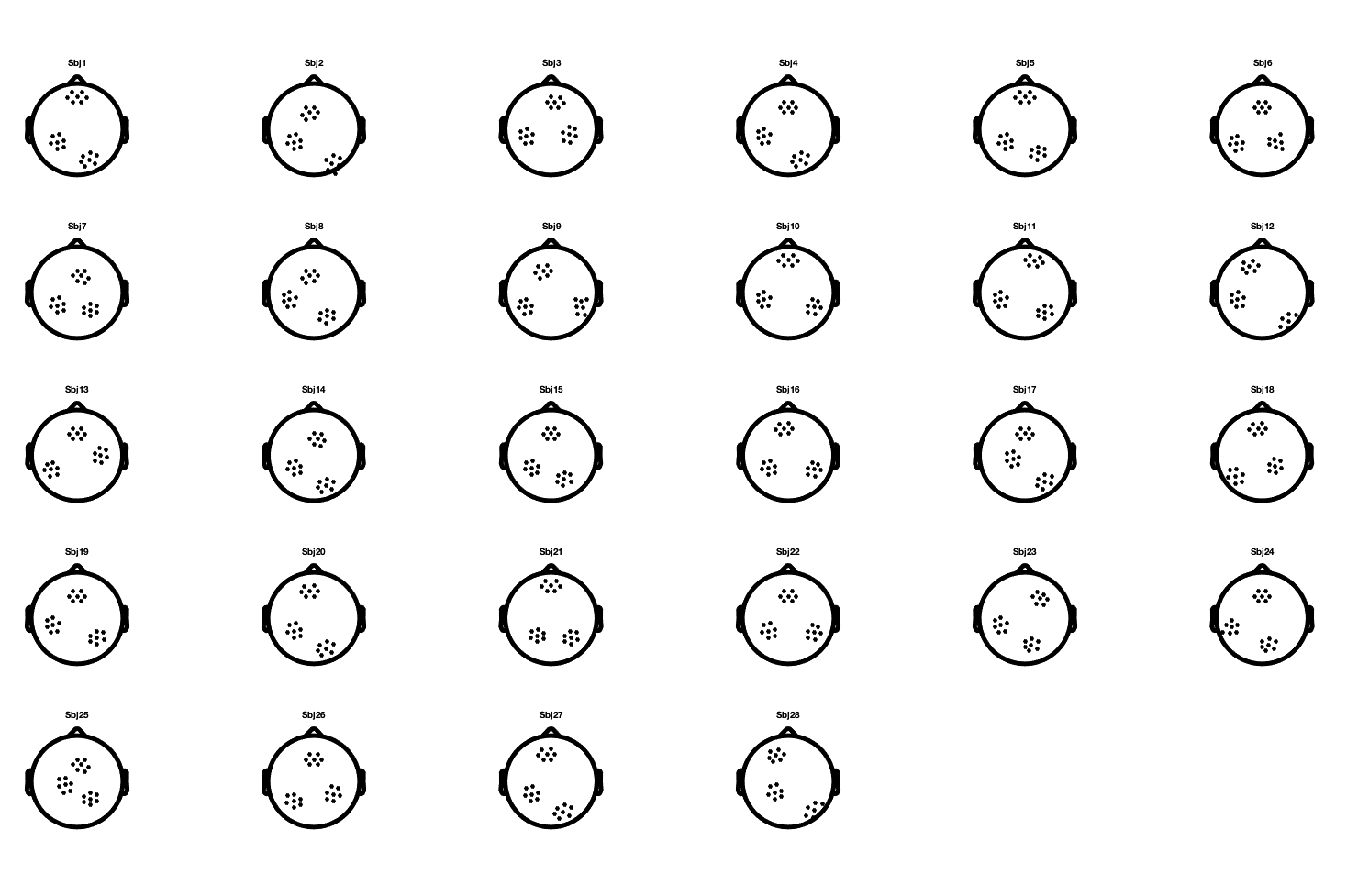

Supplement: FIGURE S1 — Topographies showing the selected channels for the Left, Frontal, and Right ROIs for each subject in the ROT group. [file Image_1.TIF]

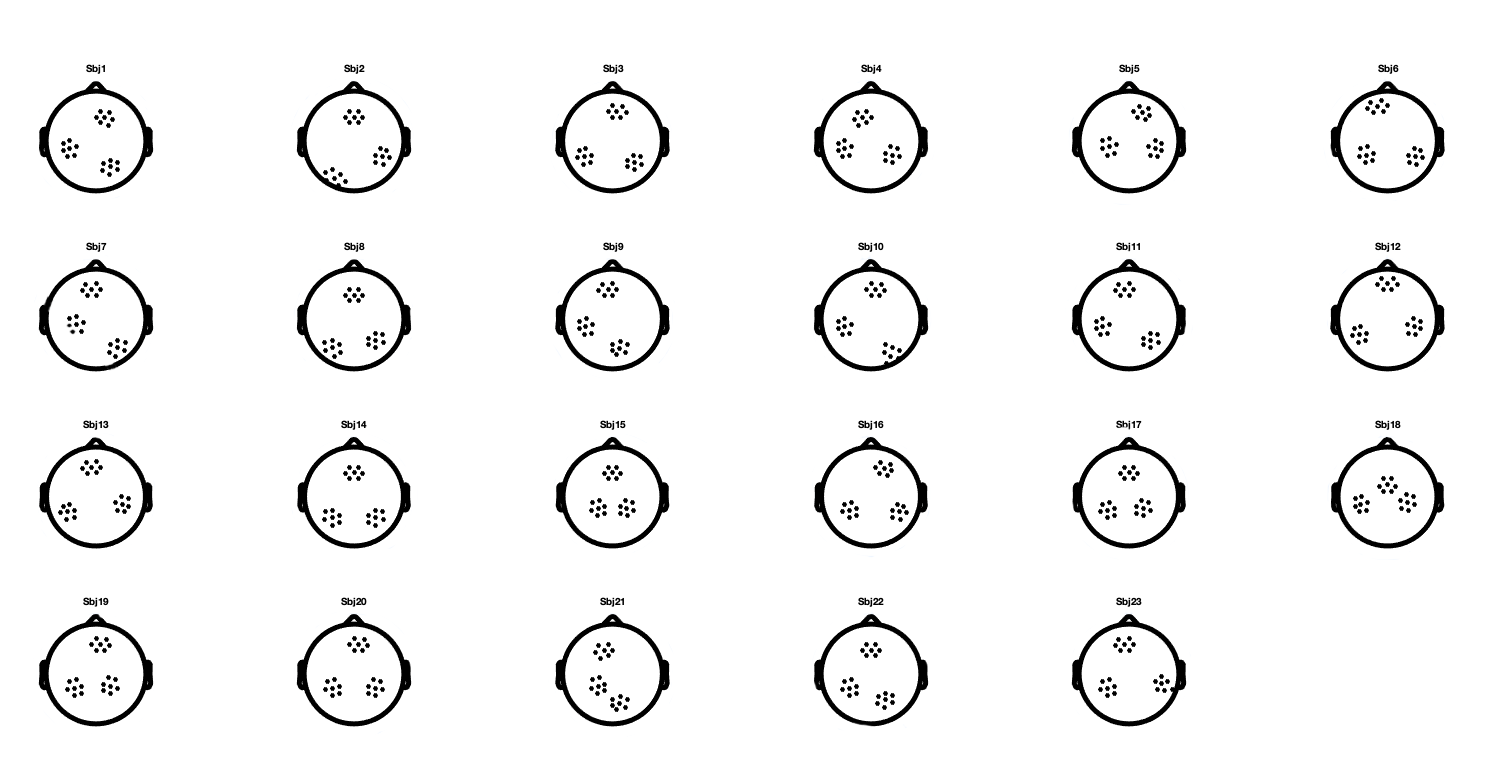

Supplement: FIGURE S2 — Topographies showing the selected channels for the Left, Frontal, and Right ROIs for each subject in the VSEQ group. [file Image_2.TIF]

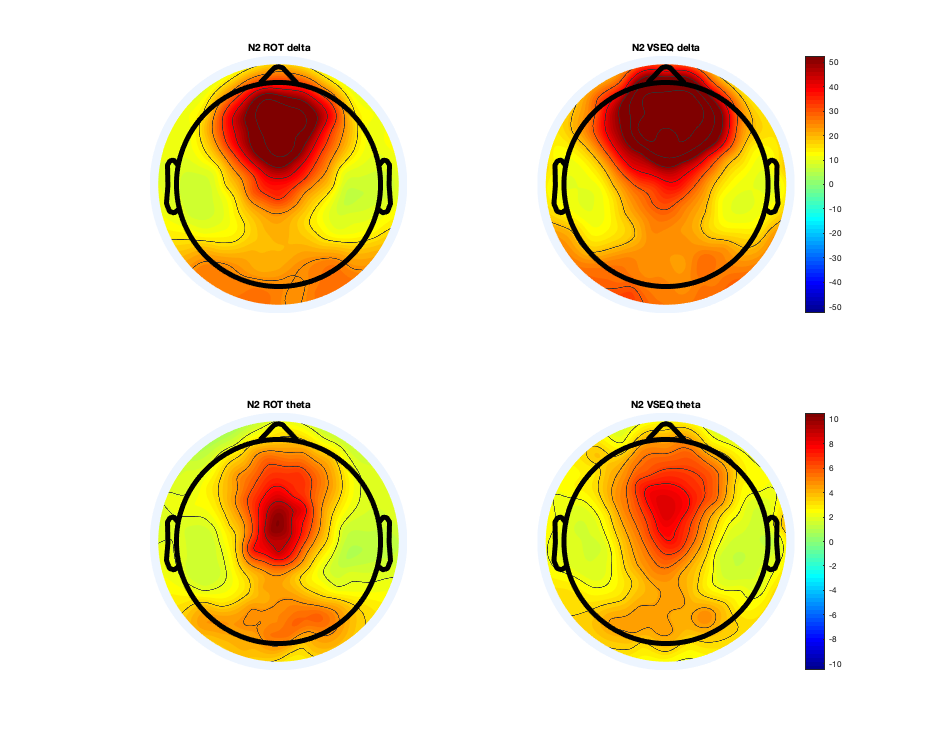

Supplement: FIGURE S3 — Delta and theta topographies during N2 and N3 stages in the ROT and VSEQ groups. [file Image_3.TIF]
