# Supplementary material for: Prior Practice Affects Movement-Related Beta Modulation and Quiet Wake Restores It to Baseline
Source: Front Syst Neurosci. 2020 Aug 18;14:61. doi: 10.3389/fnsys.2020.00061 (PMC7462015; doi:10.3389/fnsys.2020.00061)
Supplement: TABLE S1 — Results of mixed model ANOVAs for the delta and theta amplitudes in the selected ROIs during N2 and N3 stages in the ROT and VSEQ Nap groups. [file Table_1.docx]

|  |  |  |  |  |  |  |  |
| --- | --- | --- | --- | --- | --- | --- | --- |
|  |  |  | **df** | **F** | **p** | **η2p** |  |
|  | **Delta N2** | ROI | 1.0, 18.6 | 32.9 | **<0.001** | 0.646 |  |
|  |  | ROI x Group | 1.0, 18.6 | 0.1 | 0.77 | 0.005 |  |
|  |  | Group | 1, 18 | 0.19 | 0.67 | 0.01 |  |
|  | **Theta N2** | ROI | 2, 36 | 29.36 | **<0.001** | 0.62 |  |
|  |  | ROI x Group | 2, 36 | 0.22 | 0.8 | 0.012 |  |
|  |  | Group | 1, 18 | 0.001 | 0.97 | 0.000067 |  |
|  | **Delta N3** | ROI | 1.2, 16.15 | 30.22 | **<0.001** | 0.699 |  |
|  |  | ROI x Group | 1.2, 16.15 | 1.12 | 0.32 | 0.079 |  |
|  |  | Group | 1, 13 | 1.17 | 0.3 | 0.083 |  |
|  | **Theta N3** | ROI | 1.4, 18.4 | 37.92 | **<0.001** | 0.745 |  |
|  |  | ROI x Group | 1.4, 18.4 | 0.08 | 0.86 | 0.006 |  |
|  |  | Group | 1, 13 | 0.02 | 0.91 | 0.001 |  |
|  |  |  |  |  |  |  |  |
|  |  |  | **ROT-VSEQ** | **Left-Front** | **Left-Right** | **Right-Front** |  |
|  | **Delta N2** | Mean diff | -6.432 | -93.714 | -3.031 | -90.683 |  |
|  |  | SE | 14.846 | 16.551 | 2.649 | 15.369 |  |
|  |  | p | 0.67 | **<0.001** | 0.802 | **<0.001** |  |
|  | **Theta N2** | Mean diff | -0.059 | -5.994 | -0.452 | -5.542 |  |
|  |  | SE | 1.696 | 0.991 | 0.78 | 0.828 |  |
|  |  | p | 0.973 | **<0.001** | 1 | **<0.001** |  |
|  | **Delta N3** | Mean diff | -39.599 | -171.938 | -13.332 | -158.605 |  |
|  |  | SE | 36.619 | 29.269 | 11.536 | 28.75 |  |
|  |  | p | 0.299 | **<0.001** | 0.806 | **<0.001** |  |
|  | **Theta N3** | Mean diff | 0.275 | -9.373 | -0.432 | -8.941 |  |
|  |  | SE | 2.278 | 1.38 | 0.728 | 1.413 |  |
|  |  | p | 0.906 | **<0.001** | 1 | **<0.001** |  |
|  |  |  |  |  |  |  |  |

**Supplemental Table 1**
