# Supplementary material for: Prior Practice Affects Movement-Related Beta Modulation and Quiet Wake Restores It to Baseline
Source: Front Syst Neurosci. 2020 Aug 18;14:61. doi: 10.3389/fnsys.2020.00061 (PMC7462015; doi:10.3389/fnsys.2020.00061)
Supplement: Supplementary file 5 [file Data_Sheet_1.pdf]

Supplemental material

Methods

Nap recordings of both ROT and VSEQ groups were preprocessed with the same procedure described in the Methods section. Eleven and nine subjects were included for ROT and VSEQ respectively; the others were rejected for technical problems in either recording or storage. Spectral analysis (epoch length: 4s, Multitaper Fast Fourier Transform, Hanning taper, 4 cycles adaptive window width, 0.5Hz frequency step) was run on the trials corresponding to stages N1, N2, and N3 (average trials: VSEQ N1:287.62±172.96, N2: 494.62±186.25, N3: 275.12± 196.31; ROT N1:215.25±148.22, N2:417.5±145.77, N3: 405.87±233.82). Stages N2 and N3 were normalized by the total power of N1, according to the formula: $(N2 – N1_{tot\_power}) / N1_{tot\_power.}$
We extracted delta (1-4 Hz) and theta (4.5-8 Hz) amplitude during N2 (ROT Nap: 11 subjects, VSEQ Nap: 9 subjects) and N3 stages (ROT Nap: 8 subjects, VSEQ Nap: 7 subjects) from the ROIs showing the greatest beta modulation increase. Mixed-model ANOVAs with ROIs (Left, Front, and Right) as repeated-measure factor and Task, as between-subjects factor, were run to explore any condition difference during N2 and N3.

Results

As expected, both delta and theta average power during N2 and N3 was prominent over the frontal ROI (Supplemental Figure 3), resulting in a significant main effect of ROIs (Supplemental Table 1), with no ROIs x Task interaction. No main effect was observed between VSEQ and ROT nap subgroups. However, the small sample size of these analyses reduces the strength of the evidences about the lack of a Task effect; therefore, any conclusion in this regard should be considered cautiously.
